# Supplementary material for: BMP suppresses Wnt signaling via the Bcl11b-regulated NuRD complex to maintain intestinal stem cells
Source: EMBO J. 2024 Oct 21;43(23):6032–51. doi: 10.1038/s44318-024-00276-1 (PMC11612440; doi:10.1038/s44318-024-00276-1)
Supplement: Supplementary file 1 — Appendix [file 44318_2024_276_MOESM1_ESM.pdf]

Appendix

BMP suppresses Wnt signaling via the Bcl11b-regulated NuRD complex to maintain intestinal stem cells

Yehua Li[1,†], Xiaodan Wang[1,†], Meimei Huang[1,†], Xu Wang[2], Chunlin Li[1], Siqi Li[2], Yuhui Tang[1], Shicheng Yu[2], Yalong Wang[2], Wanglu Song[1], Wei Wu[3], Yuan Liu[1,*], Ye-Guang Chen[1,2,4,*]

Table of contents:

Appendix Figure S1: Bcl11b maintains Lgr5$^+$ ISC Page-2-4

Appendix Figure S2: Bcl11b deletion impedes regeneration of intestinal epithelium after injury Page-5

Appendix Figure S3: Bcl11b promotes Wnt/β-catenin signaling Page-6-7

Appendix Figure S4: Bcl11b interacts with TCF4 and β-catenin Page-8

Appendix Figure S5: Bcl11b interacts with the NuRD complex Page-9

Appendix Figure S6: Bcl11b affects the chromatin dynamics Page-10

Appendix Figure S7: Bcl11b is upregulated in the colorectal cancer and promotes tumor Page-11-12

Appendix Table S1: PCR primer sequence Page-13-14

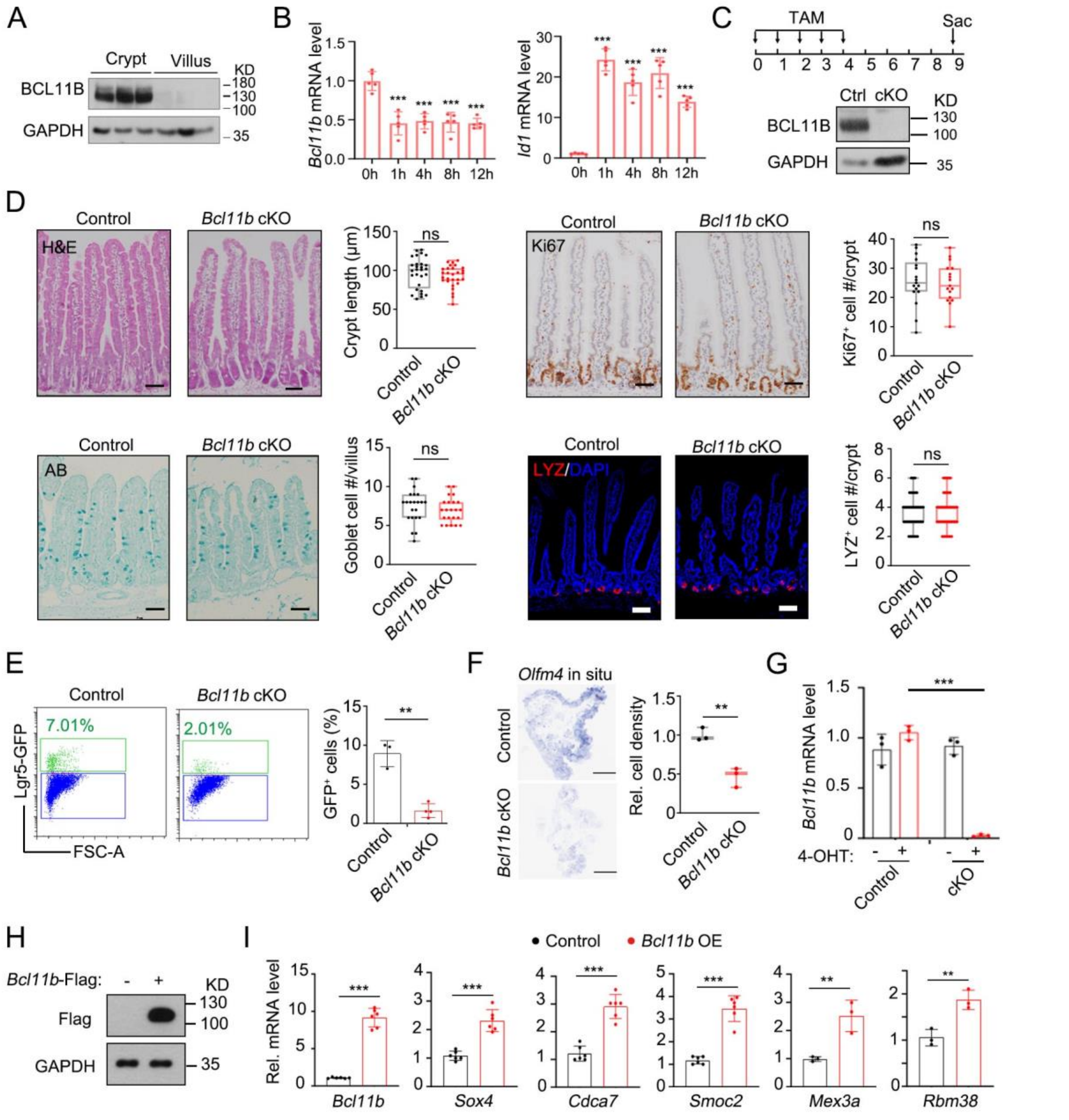

Appendix Figure S1. Bcl11b maintains Lgr5^{+} ISC

(A) Anti-BCL11B immunoblotting was performed with the isolated crypts and villus of wild-type mice. n=3 mice/group. (B) Organoids derived from the crypts of WT mice were treated with ERB for indicated times. Then, the organoids were harvested for analysis of *Bcl11b* and *Id1* by qRT-PCR. E, EGF, 50 ng/mL; R, R-spondin 1, 500 ng/mL; B, BMP4, 20 ng/mL. n=5 independent experiments. (C) Schematic illustration of the experimental strategy of *Bcl11b*

knockout in mice (top). Anti-Bcl11b immunoblotting was performed with the crypts from control and *Bcl11b* cKO mice (bottom). (D) *Vil-CreERT2;Lgr5-GFP-IRES-creERT2* (control) and *Vil-CreERT2;Lgr5-GFP-IRES-creERT2;Bcl11b*$^{fl/fl}$ (Bcl11b cKO) mice were treated as in (C), then H&E, Ki67 and Alcian blue (AB) staining of small intestinal sections were performed at day 9. Lysozyme (LYZ) staining was performed 4 weeks after injection. The graph depicts the number of ≥ 17 crypts or villus measured on tissue sections from n=3 mice/group. Scale bar, 50 μm. (E) Cells isolated from the crypts of *Vil-CreERT2;Lgr5-GFP-IRES-creERT2* (control) and *Vil-CreERT2;Lgr5-GFP-IRES-creERT2;Bcl11b*$^{fl/fl}$ (*Bcl11b* cKO) mice were subjected to FACS analysis for GFP-high cells. Left panel shows a representative FACS result. Right panel shows the percentage of GFP$^+$ cells. n=3 mice/group. Each dot represents one mouse. (F) Organoids derived from the crypts of *Vil-CreERT2;Lgr5-GFP-IRES-creERT2* (control) and *Vil-CreERT2;Lgr5-GFP-IRES-creERT2;Bcl11b*$^{fl/fl}$ (*Bcl11b* cKO) mice were treated with 4-OHT for 48h and were fixed to do paraffin sections 48h later. Then *in situ* hybridization was performed to examine *Olfm4* expression. n= three independent experiments. Each dot represents one experiment. (G) Lgr5$^+$ ISCs of *Vil-CreERT2;Lgr5-GFP-IRES-creERT2* (control) and *Vil-CreERT2;Lgr5-GFP-IRES-creERT2;Bcl11b*$^{fl/fl}$ (*Bcl11b* cKO) mice were sorted by FACS. About 5000 cells were seeded in each well for single cell culture upon 4-OHT treatment. Six days later, the expression of *Bcl11b* was examined by q-RT-PCR. n= three independent experiments. (H) Organoids derived from the crypts of *R26-M2rtTA;Vil-CreERT2* mice at day 11 after 10 μM doxycycline treatment were infected with Flag-Bcl11b-expressing lentivirus and harvested for anti-Flag immunoblotting. n= three independent experiments. (I) Organoids derived from the crypts of *R26-M2rtTA;Vil-*

CreERT2 mice at day 11 after 10 μM doxycycline treatment were infected with Flag-Bcl11b-expressing lentivirus and harvested for qRT-PCR. Data represent mean±sd. n= six independent experiments (*Bcl11b*, *Sox4*, *Cdca7*, *Smoc2*) and three independent experiments (*Mex3a* and *Rbm38*). The statistical analysis was performed by unpaired t test with Welch-correction (D-F, I) and two-way ANOVA with Tukey's multiple comparisons test (G). ns, not significant. **P<0.01 and ***P<0.001.

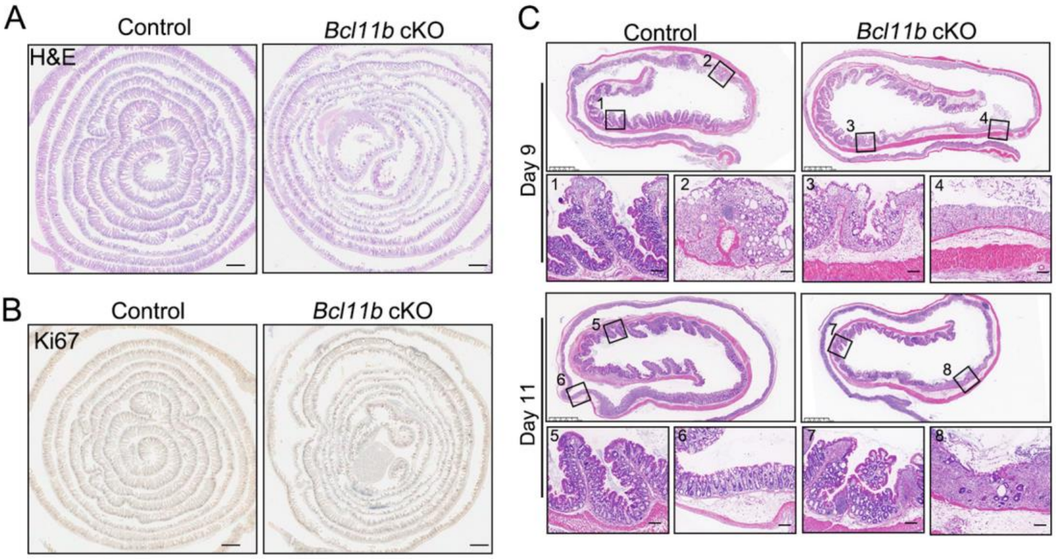

Appendix Figure S2. ***Bcl11b*** **deletion impedes regeneration of intestinal epithelium after injury**

(A and B) *Vil-CreERT2;Lgr5-GFP-IRES-creERT2* (control) and *Vil-CreERT2;Lgr5-GFP-IRES-creERT2;Bcl11b*$^{fl/fl}$ (*Bcl11b* cKO) mice were treated with tamoxifen for 5 consecutive days, exposed to 10 Gy X-ray radiation 2 days later and sacrificed 5 days later for analysis. H&E staining (A) or Ki67 staining (B) were shown. Scale bar, 800 μm. (C) H&E staining from *Vil-CreERT2;Lgr5-GFP-IRES-creERT2* (control) and *Vil-CreERT2;Lgr5-GFP-IRES-creERT2;Bcl11b*$^{fl/fl}$ (*Bcl11b* cKO) mice with or without DSS treatment at the indicated time. Scale bar, 100 μm.

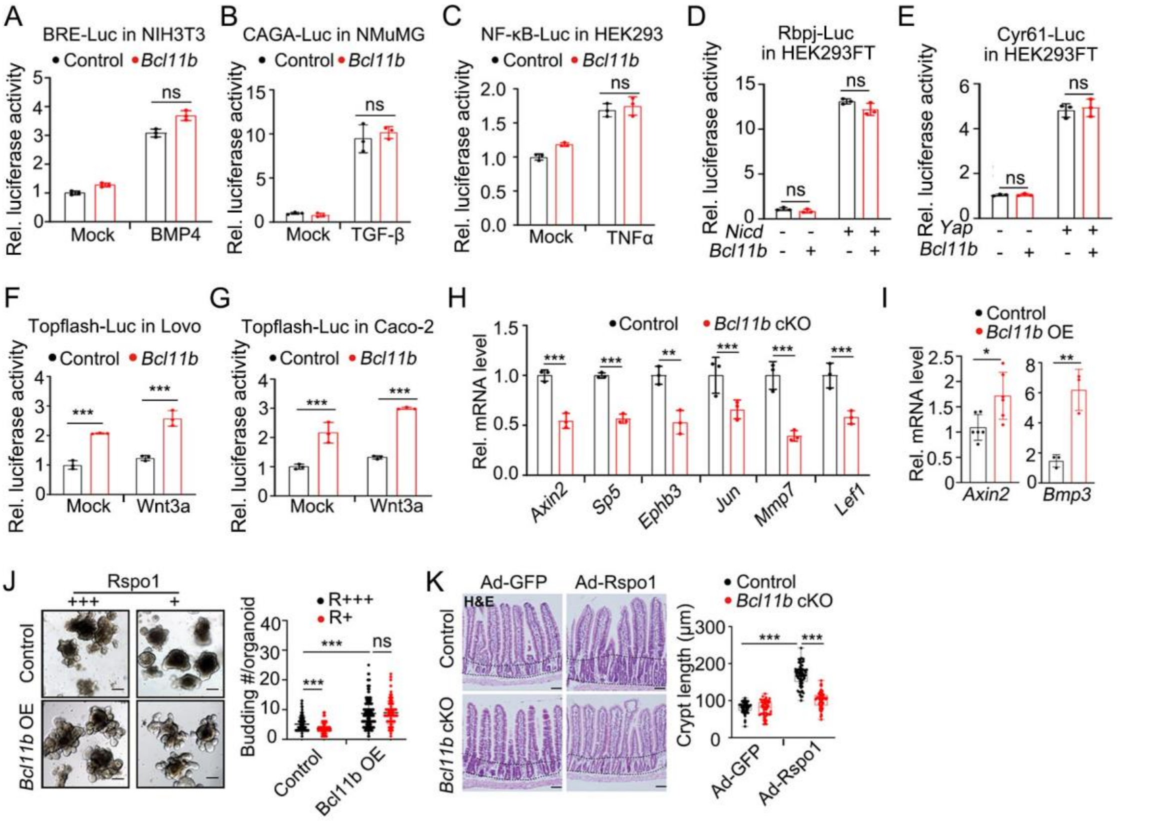

Appendix Figure S3. **Bcl11b promotes Wnt/β-catenin signaling**

(A-E) The effect of *Bcl11b* on BMP (A), TGF-β (B), NF-κB (C), Notch (D) and Hippo (E) signaling were examined by reporter assay in indicated cell lines. Cells were transfected with the indicated reporter and then treated with the indicated ligand for 18h before harvested for luciferase activity. (F and G) Colon cancer cells LoVo (F) and Caco-2 (G) were transfected with empty or *Bcl11b* plasmid together with Topflash-luciferase or Fopflash-luciferase, and then treated with 20 ng/mL Wnt-3a for 18h before harvested for luciferase measurement. The ratio of Topflash-luciferase to Fopflash-luciferase was shown. (H) Organoids derived from small intestinal crypts of *Vil-CreERT2;Lgr5-GFP-IRES-creERT2* (control) and *Vil-CreERT2;Lgr5-GFP-IRES-creERT2;Bcl11b*$^{fl/fl}$ (*Bcl11b* cKO) mice were treated with 4-OHT for 48h, and then mRNA was extracted for qRT-PCR. n= three independent experiments. (I) Organoids derived from the crypts of *R26-M2rtTA;Vil-CreERT2* mice at day 11 after 10 μM

doxycycline treatment were infected with Flag-Bcl11b-expressing lentivirus and examined for the expression of *Axin2* and *Bmp3*. n= six independent experiments (*Axin2*) and three independent experiments (*Bmp3*). (J) Organoids derived from the crypts of *R26-M2rtTA;Vil-CreERT2* mice, which treated with 10 μM doxycycline for 9 days and with different amount of R-spondin 1 (Rspo-1) for last 3 days, were infected with Flag-Bcl11b-expressing lentivirus. Representative morphology images (left) and budding number (right) were shown. +++ represented for 500 ng/mL and + for 100 ng/mL. Scale bar, 100 μm. n= three independent experiments. Each dot represents one organoid. At least 80 organoids were measured from three independent experiments. (K) *Vil-CreERT2;Lgr5-GFP-IRES-creERT2* (control) and *Vil-CreERT2;Lgr5-GFP-IRES-creERT2;Bcl11b*$^{fl/fl}$ (*Bcl11b* cKO) mice were injected adenovirus expressing GFP or Rspo-1 one day after 5 times daily tamoxifen administration. The mice were sacrificed 5 days later for H&E staining of the intestinal epithelium. Representative image (left) and quantification (right) are shown. The dashed lines mark the crypt region of the intestinal epithelium. Scale bar, 100 μm. n=3 mice/group. Each dot represents one crypt. At least 54 crypts were measured from three mice. Data represent mean±sd. The data were analyzed by unpaired t test with Welch-correction (I) and two-way ANOVA with Tukey's multiple comparisons test (A-H and J, K). ns, not significant. *P<0.05, **P<0.01 and ***P<0.001.

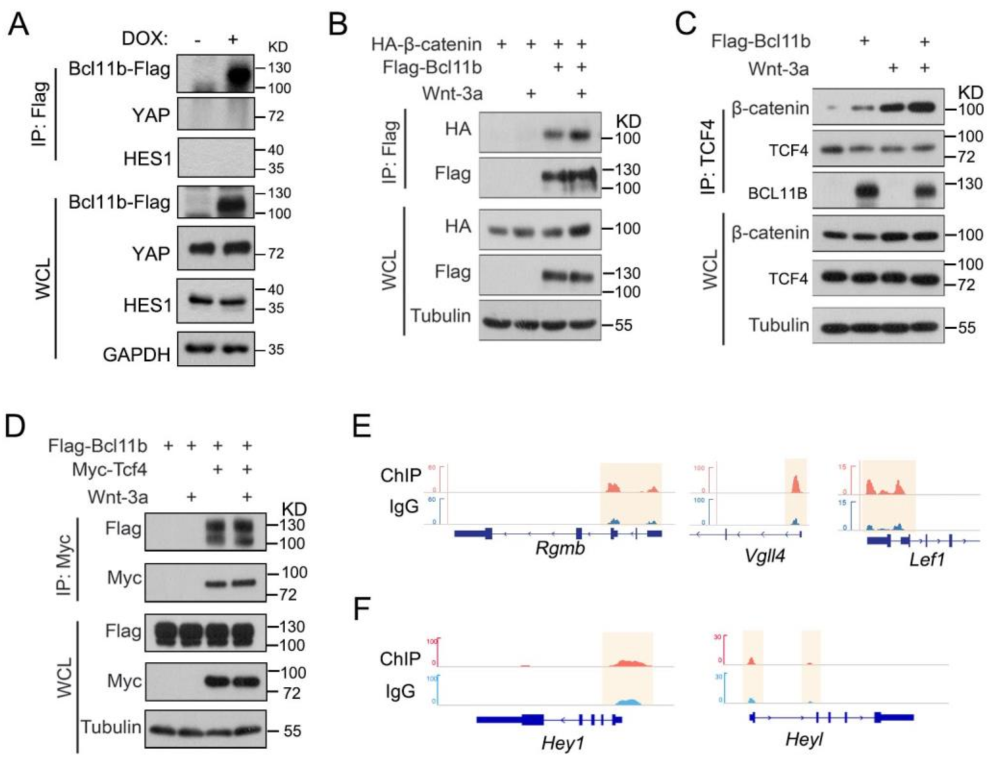

Appendix Figure S4. Bcl11b interacts with TCF4 and β-catenin

(A) Organoids derived from the crypts of *R26-M2rtTA;Vil-CreERT2* mice were infected with *Bcl11b*-expressing lentivirus, treated with doxycycline for 3 days and then harvested for immunoprecipitation with anti-Flag antibody and immunoblotting with indicated antibodies. (B-D) HEK293T cells were transfected with indicated plasmids. Wnt-3a was added 36h later and cells were harvested for immunoprecipitation and immunoblotting with indicated antibodies 12h later. Protein expression was confirmed by immunoblotting of total cell lysates. (E and F) Integrative Genomics Viewer (IGV) tracks displaying ChIP-seq reads along the indicated genes in intestinal organoids. Blue reads were from IgG libraries and red reads from Bcl11b-Flag-ChIP libraries. The y axis represents the CPM (count per million) of genes. The yellow boxes of the tracks depict the binding sites.

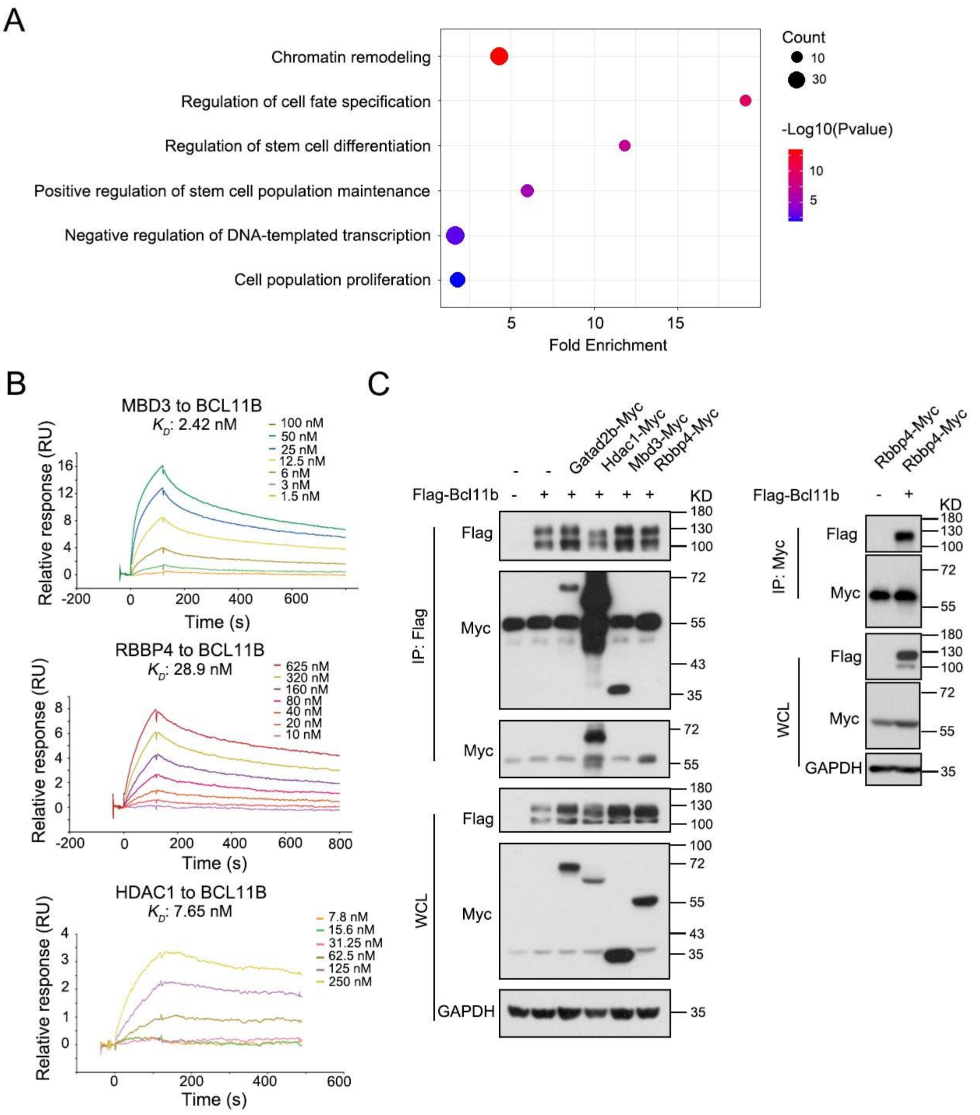

Appendix Figure S5. Bcl11b interacts with the NuRD complex

(A) Gene ontology analysis of Bcl11b-interacting proteins in biological processes by DAVID. (B) Representative SPR binding profiles of MBD3, RBBP4 and HDAC1 to BCL11B. n=three independent experiments. (C) HEK293FT cells were transfected with indicated plasmids and harvested for immunoprecipitation and immunoblotting with indicated antibodies 36h later. Protein expression was examined by immunoblotting of total cell lysates.

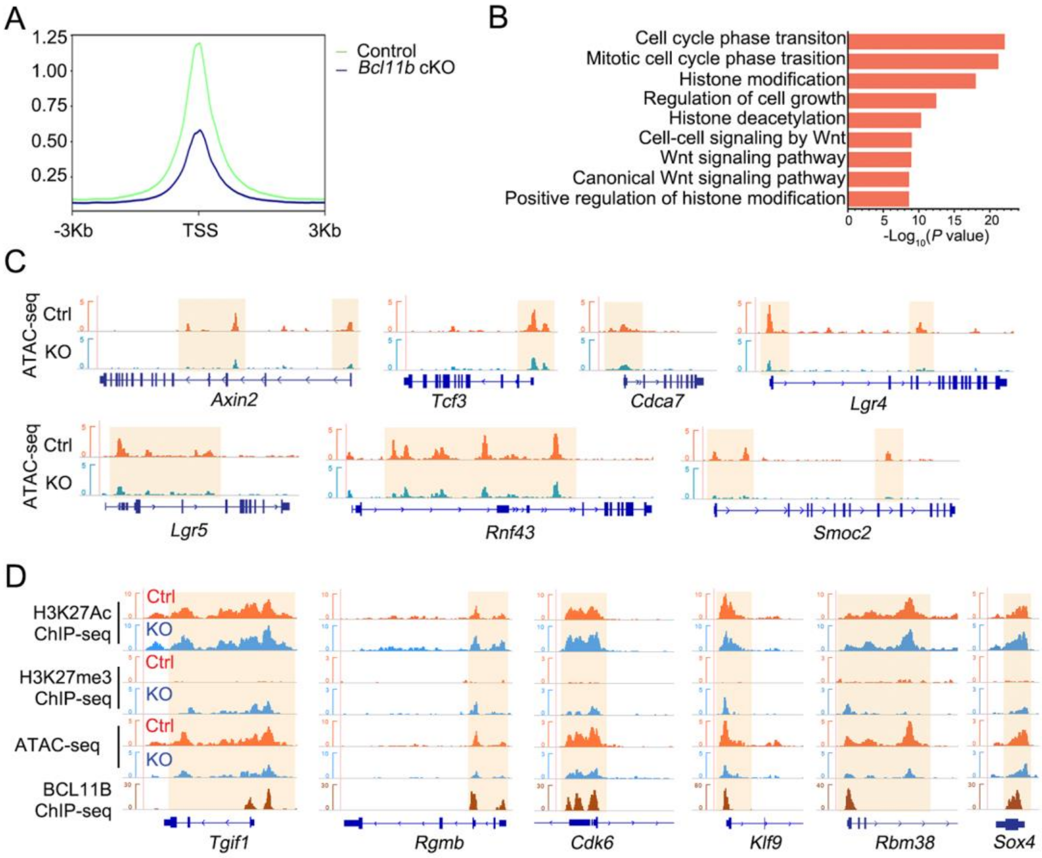

Appendix Figure S6. Bcl11b affects the chromatin dynamics

(A) Relative strengths of ATAC signals upon *Bcl11b* loss in ISCs. n=3 mice/group. (B) Functional enrichment analysis of the genes whose promoter showed lower ATAC signals in (A). (C) Integrative Genomics Viewer (IGV) tracks displaying ATAC-seq reads along the indicated genes in Lgr5^{+} ISCs from control and *Bcl11b* cKO mice. Blue reads were from KO libraries and red reads from control libraries. The y axis represents the CPM (count per million) of genes. The yellow boxes of the tracks depict the loss of signal in KO group. (D) IGV tracks displaying ChIP-seq reads and ATAC-seq reads along the indicated genes. Blue reads are from KO libraries and red reads from control libraries. The y axis represents the CPM (count per million) of genes. The yellow boxes of the tracks depict the binding sites. n=3 mice per group.

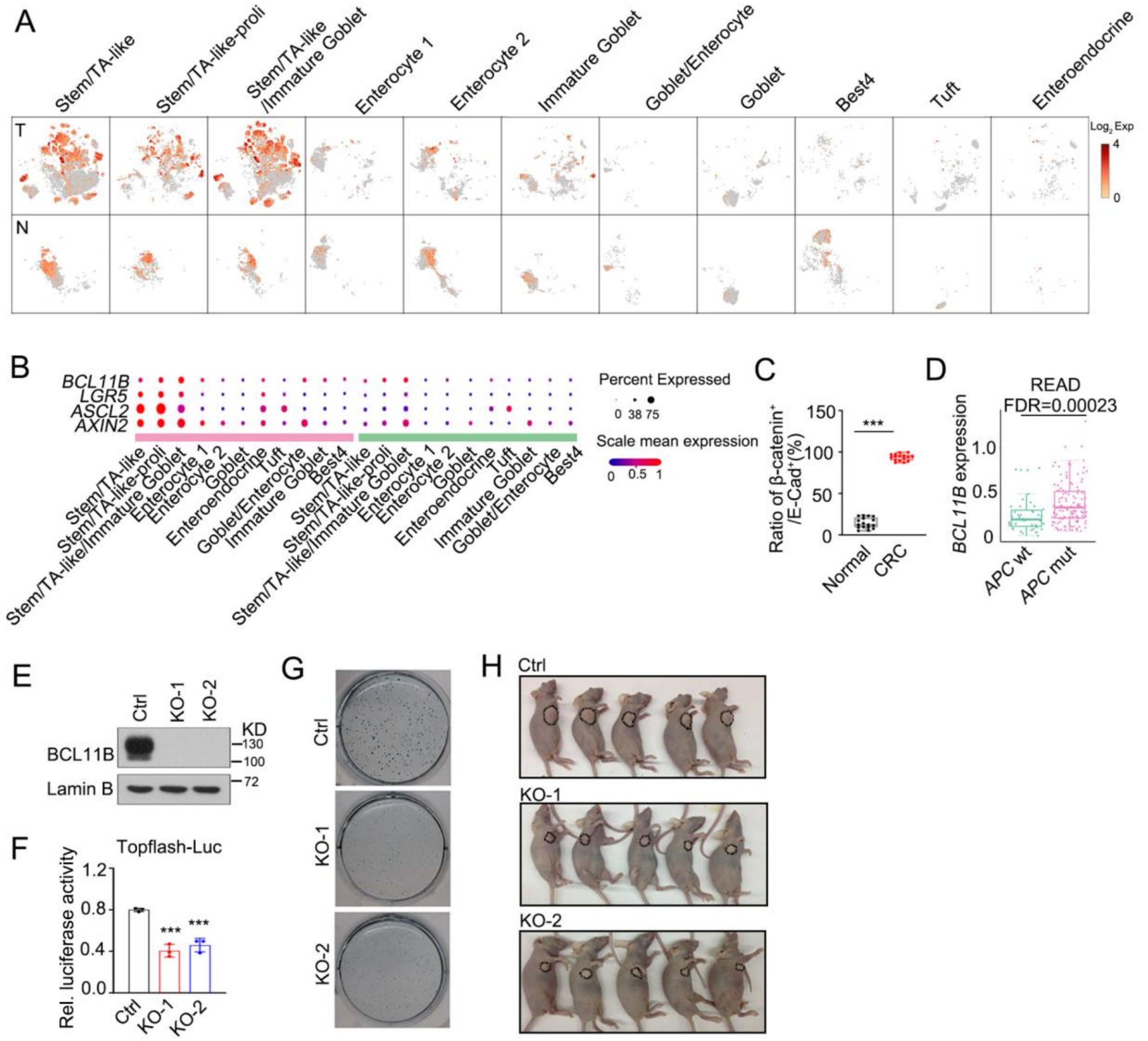

Appendix Figure S7. Bcl11b is upregulated in the colorectal cancer and promotes tumor

(A) t-SNE plot, with split on clusters, showing *BCL11B* expression in epithelial cells among different clusters from health control (Normal, N) and colorectal cancer (Tumor, T) patients based on scRNA-seq data base (GSE178341). (B) Single-cell RNA-seq analysis showing the gene expression level (color scale) and expressing cells (point diameter) in each cell cluster of health control (normal, N, green) and colorectal cancer patients (tumor, T, pink) based on scRNA-seq data base (GSE178341). (C) Quantification of nuclear β-catenin ratio in normal or CRC tissues. n=three tissues/group. Each dot represents one crypt. At least 17 crypts on

tissue sections from three different tissues. (D) The expression of *BCL11B* in READ tissues with *APC* mutation compare to wild type *APC* revealed by analysis of TCGA dataset. (E) Control and *BCL11B* knockout LoVo cells were harvested for BCL11B immunoblotting. Lamin B is a loading control of nuclear protein. (F) Control and *BCL11B* knockout LoVo cells were transfected with Topflash-luciferase or Fopflash-Luciferase. The ratio of Topflash-luciferase to Fopflash-luciferase was shown. Data were analyzed compared to control group and represented mean±sd. of three independent experiments. (G) Control and *BCL11B* knockout LoVo cells were subjected to colony formation assay. Each well was seeded with 2000 cells, and the colony numbers were counted two weeks later. n= three independent experiments. (H) Control and *BCL11B* knockout LoVo cells (10^7 cells) were subcutaneously injected into the nude mice. The mice were sacrificed 15 days later to examine the tumor size. Scale bar, 1 cm. Data represent mean±sd. The data were analyzed by unpaired t test with Welch-correction (C) and one-way ANOVA with Tukey's multiple comparisons test (F). ***P<0.001.

Appendix Table S1: PCR primer sequences

Quantitiative RT-PCR primers	forward (5'-3')	reverse (5'-3')
**m-Gapdh-rt**	AAGAAGGTGGTGAAGCAG	TCATACCAGGAAATGAGC
**m-Bmpr1a-rt**	ATGCTCCATGGCACTGGTATGA	GGCAGTGTCCTGAGCAATAGCA
**m-Id1-rt**	ATCGCATCTTGTGTCGCTGAG	AGTCTCTGGAGGCTGAAAGGT
**m-Lgr5-rt**	CGGGACCTTGAAGATTTCCT	GATTCGGATCAGCCAGCTAC
**m-Ascl2-rt**	GCCTGACCAAATGCCAAGTG	ATTTCCAAGTCCTGATGCTGC
**m-Olfm4-rt**	CGAGACTATCGGATTCGCTATG	TTGTAGGCAGCCAGAGGGAG
**m-Axin2-rt**	GCTCCAGAAGATCACAAAGAGC	AGCTTTGAGCCTTCAGCATC
**m-Bcl11b-rt5**	GATGCCCTTCAGCGTCTAC	CTCTCAGCCTGCTCGATTT
**m-Smad1/4 negative region-chip seq-rt**	GCACCATGAAGCTGGAAGAATCT	TACCCAAAGGAGCATAGCGTT
**m-Bcl11b-chip-seq1-rt1**	TCACCATGCCTGACCTAAAG	CCATAGGTGTGTGGGTTCAT
**m-Bcl11b-chip-seq1-rt2**	TCATGGATTGGCAGGATCAA	GTGATTCCACCAGAGGTTCTT
**m-Smoc2-rt**	CCCAAGCTCCCCTCAGAAG	GCCACACACCTGGACACAT
**m-Sox4-rt**	ATGAACGCCTTTATGGTGTGG	TCCTGGATGAACGGAATCTTGT
**m-Pdgfa-rt**	GTAACACCAGCAGCGTCAAGT	TGGCTTCTTCCTGACATACTCC
**m-Cdk6-rt**	GGCGTACCCACAGAAACCATA	AGGTAAGGGCCATCTGAAAACT
**m-Cdca7-rt**	ATGTCATCAGTGTCGCCAGAAA	CCTCGCCATAGCGGTTTCG
**m-Pla2g4a-rt**	GCATTTCTTGATGGGTGTCTGG	AGCCTCATCATCACTGTCGGA
**m-Agr3-rt**	TTCAAACCTTGCCATTGCTAT	TGGTTTGTTACTTTTGCGAGC
**m-Clca4-rt**	GAAAGAACACTGTCGAAGCAAC	CAGTCTGGGATTTGTTTGGGATA
**m-Rnf32-rt**	GTTGCCTTACAGGACCACCTA	CTATGATGGCTGTTGCATCTCTT
**m-Dtx4-rt**	GCTGGCTTCTTGCTGCTTCC	CAGTCTACACCTCTCTCCCCAA
**m-Rgmb-rt**	CACGGATCAGAAGGTGTACCA	AGCTGTCGCACAAACACTGT
**m-Sp5-rt**	CATCGAGGTAGCTGACAAAGAG	CTCAACTACGACCCAGTTTCC
**m-RNF43-rt2**	GATCACGTCTCACCTCCTTTC	GGCTCACCTAAACTCCTTCTTC

**m-Sox9-rt**	GCCAGATGGACCCACCAGTAT	TCCAAACAGGCAGGGAGATTC
**m-Mmp7-rt**	GAGTGCCAGATGTTGCAGAATA	ATCCACTACGATCCGAGGTAAG
**m-Jun-rt**	CCAGACTGTACACCAGAAGATG	CAACCAAAGTGTCTGCTTTCC
**m-Lef1-rt**	AGAACACCCTGATGAAGGAAAG	GTACGGGTCGCTGTTCATATT
**m-EphB3-rt1**	CATGGACACGAAATGGGTGAC	GCGGATAGGATTCATGGCTTCA
**m-Tnfrsf19-rt**	CTTCCGTGACAGCATTTACCTT	CTGCTCAGTGAAGCCATAGGG
**m-Bmp3-rt**	GATGAGCAGACCCTGAAGAAG	TCCAGCCGATATCAGCAAAG
**m-Tgif1-chip-rt1**	AAGCAGGCAGGAACCTTAAA	TGGACACTGTCGTGCAAC
**m-Tgif1-chip-rt2**	TTGCAGAGTGCTCCCTAAGT	TTGGGTTCTCCCTGGACAC
**m-Sox4-chip-rt1**	TCACCGCTGAAGTGCTC	GAAGATACTCCGGCTACCAAC
**m-Sox4-chip-rt2**	GCCTCCATCTTCGTACAACC	CCTGCCGACAAGAAAGTGAA
**m-Sox4-chip-rt3**	CAGGCTTCCAAACAGGTAGA	GCTGCATCGTTCTCTCCA
**m-Fzd7-chip-rt1**	TTCCGTGCAACTCCGTTT	AATGGCTCGTTCTCGCTTT
**m-Fzd7-chip-rt2**	CCAGCTTGTCCAGCTCTTAC	TCCTTCAGTCCTAGGAAGGTC
**m-Klf9-chip-rt**	TGTGACGTGATGGGATTCTG	CGCCTACGAGACACTTCTTC
**m-Mex3a-chip-rt**	CAAGGGAGCGACCATCAAA	GTGCGCCGGTTATCTCAA
**m-Rbm38-chip-rt1**	GACACCACGTTCACCAAGA	TCCGAAGCCCTCGAAGTA
**m-Rbm38-chip-rt2**	CACAGAAGGACACCACGTT	AGTACTTTCTGAGCGATGCG
**m-Rgmb-chip-rt**	CAGTAAACACTCCTGCTGAGA	TCTCGGGTTCACGTTCCT
**m-Sox9-chip-rt1**	GGGCTCGCGTATGAATCT	AGTCCTCCGACATGGTG
**m-Sox9-chip-rt2**	GACCAGTACCCGCATCT	CCCAGGATGCACAACTC
**m-Tbx3-chip-rt**	TATTGGCCGAAAGAGAGGTTC	GCTTCTCTTGTGCGGATCT
**m-Vgll4-chip-rt**	CACGGCATTTCTCAGCATTC	CACCTGGACACGCATCA
