# Supplementary figures and images for: BMP suppresses Wnt signaling via the Bcl11b-regulated NuRD complex to maintain intestinal stem cells
Source: EMBO J. 2024 Oct 21;43(23):6032–51. doi: 10.1038/s44318-024-00276-1 (PMC11612440; doi:10.1038/s44318-024-00276-1)

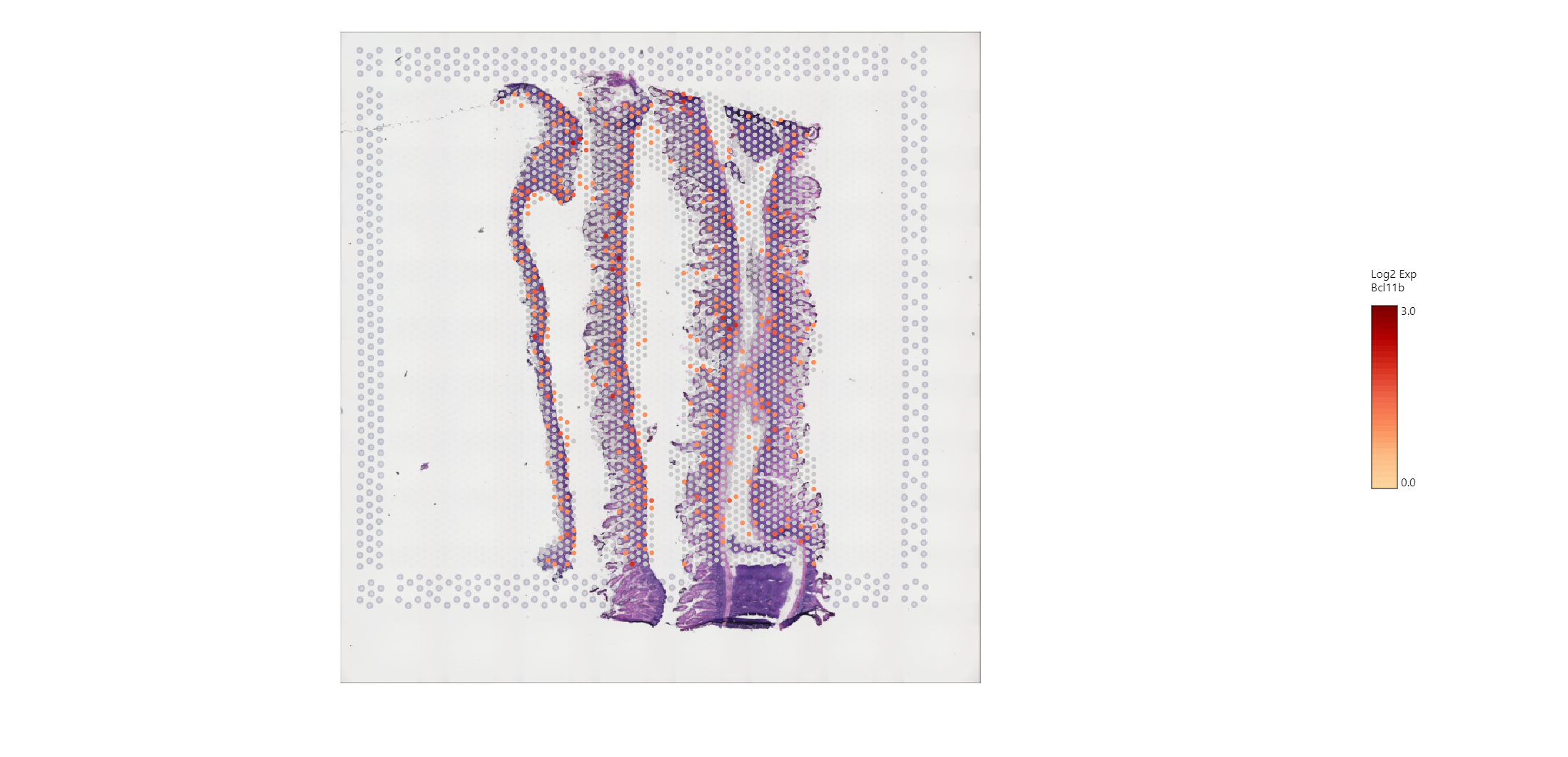

Supplement: Supplementary file 6 — Source data Fig. 1 [file 44318_2024_276_MOESM6_ESM.zip › Figure 1/1D/Bcl11b.png]

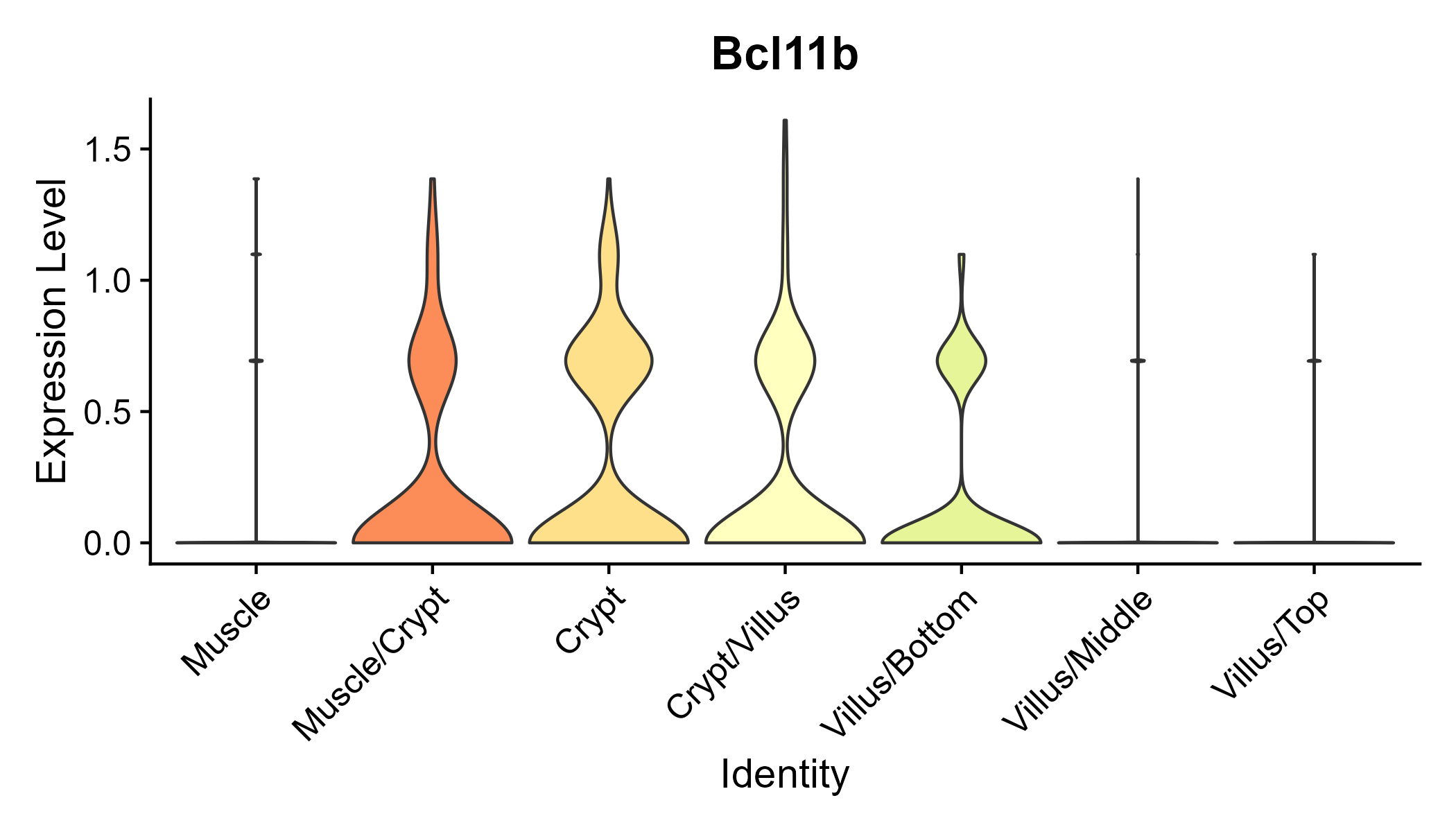

Supplement: Supplementary file 6 — Source data Fig. 1 [file 44318_2024_276_MOESM6_ESM.zip › Figure 1/1E/1E.tiff]

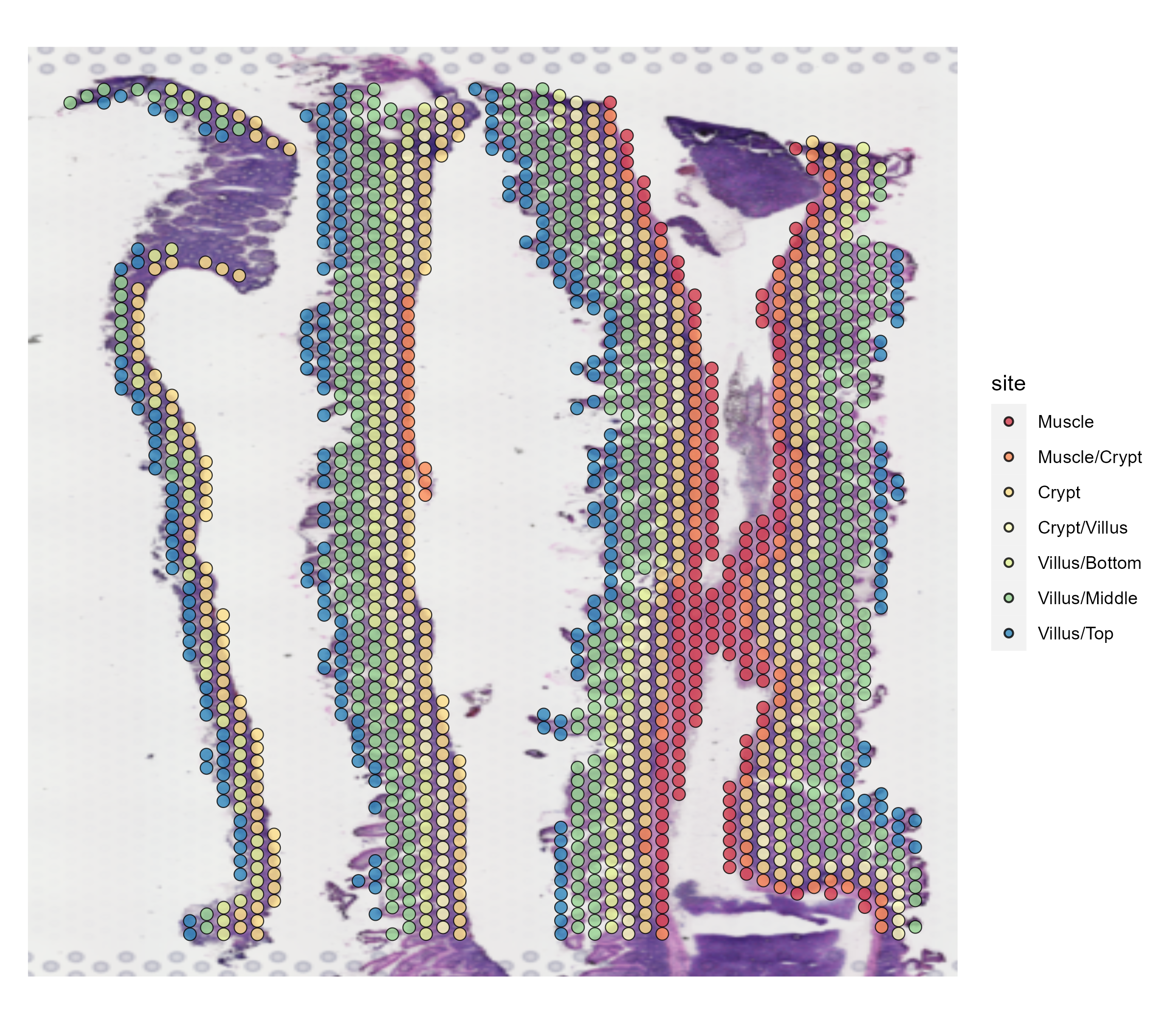

Supplement: Supplementary file 6 — Source data Fig. 1 [file 44318_2024_276_MOESM6_ESM.zip › Figure 1/1E/image-1E.tiff]

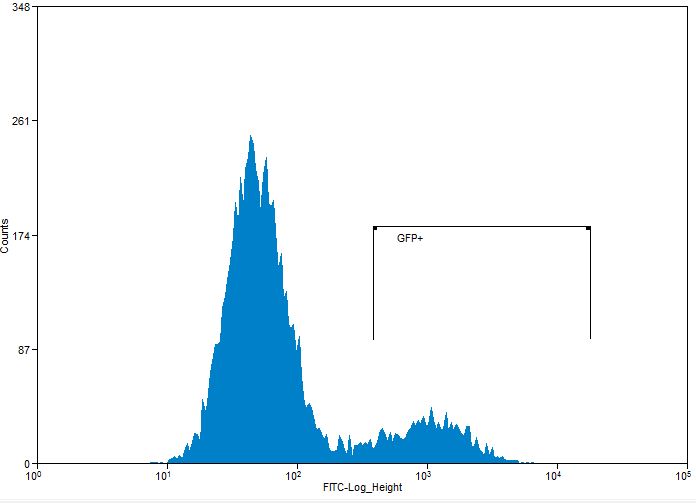

Supplement: Supplementary file 7 — Source data Fig. 2 [file 44318_2024_276_MOESM7_ESM.zip › Figure 2/2D/FACS-Bcl cKO-ETOH-1-representive.JPG]

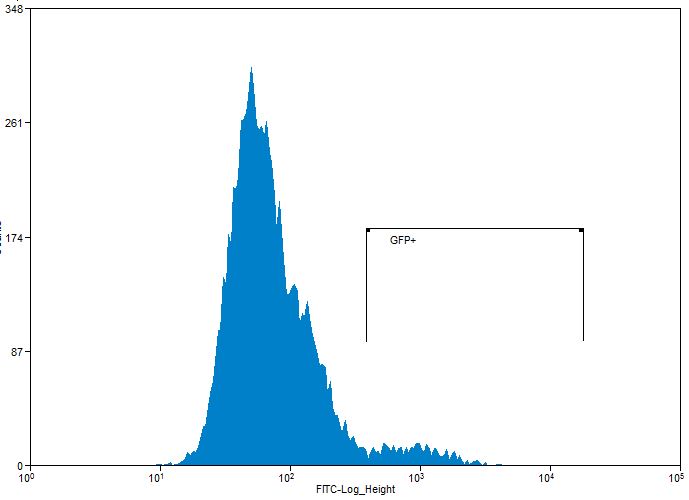

Supplement: Supplementary file 7 — Source data Fig. 2 [file 44318_2024_276_MOESM7_ESM.zip › Figure 2/2D/FACS-Bcl KO-4OHT-1-representive.JPG]

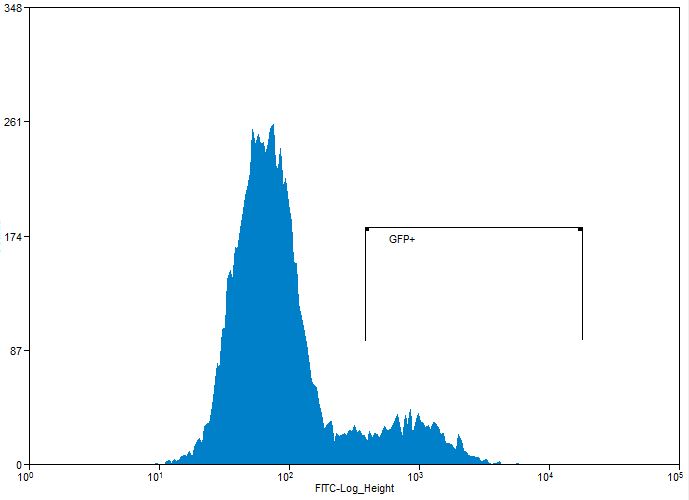

Supplement: Supplementary file 7 — Source data Fig. 2 [file 44318_2024_276_MOESM7_ESM.zip › Figure 2/2D/FACS-control-4OHT-1--representive.JPG]

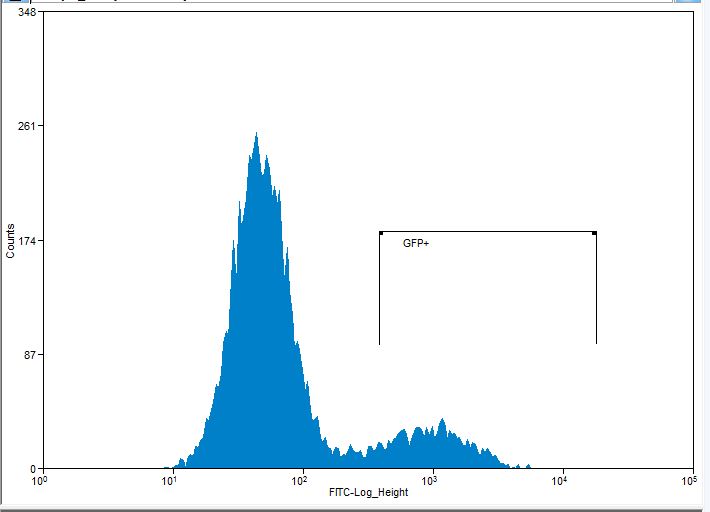

Supplement: Supplementary file 7 — Source data Fig. 2 [file 44318_2024_276_MOESM7_ESM.zip › Figure 2/2D/FACS-control-ETOH-1-representive.JPG]

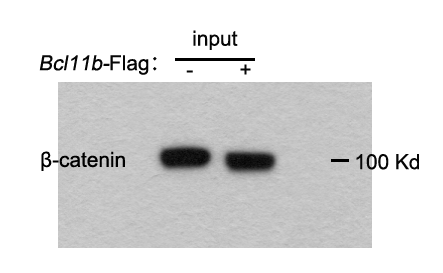

Supplement: Supplementary file 9 — Source data Fig. 4 [file 44318_2024_276_MOESM9_ESM.zip › Figure 4/4F/b-catenin-input.tif]

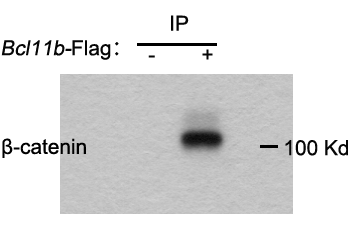

Supplement: Supplementary file 9 — Source data Fig. 4 [file 44318_2024_276_MOESM9_ESM.zip › Figure 4/4F/b-catenin-ip.tif]

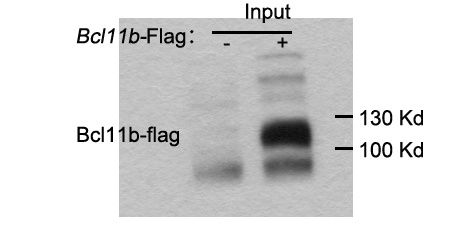

Supplement: Supplementary file 9 — Source data Fig. 4 [file 44318_2024_276_MOESM9_ESM.zip › Figure 4/4F/bcl11b-flag-input.tif]

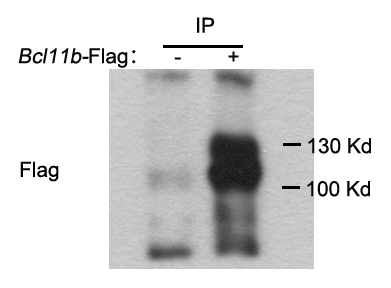

Supplement: Supplementary file 9 — Source data Fig. 4 [file 44318_2024_276_MOESM9_ESM.zip › Figure 4/4F/bcl11b-flag-IP.tif]

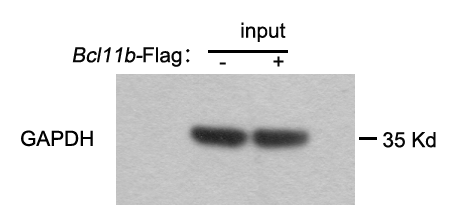

Supplement: Supplementary file 9 — Source data Fig. 4 [file 44318_2024_276_MOESM9_ESM.zip › Figure 4/4F/gapdh-input.tif]

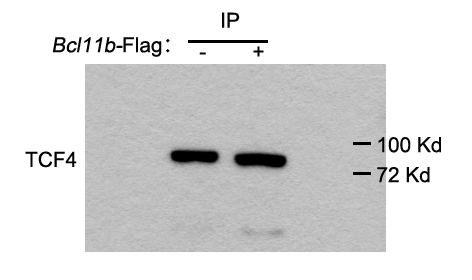

Supplement: Supplementary file 9 — Source data Fig. 4 [file 44318_2024_276_MOESM9_ESM.zip › Figure 4/4F/TCF4-input.tif]

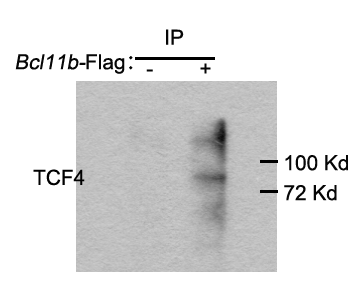

Supplement: Supplementary file 9 — Source data Fig. 4 [file 44318_2024_276_MOESM9_ESM.zip › Figure 4/4F/TCF4-IP.tif]

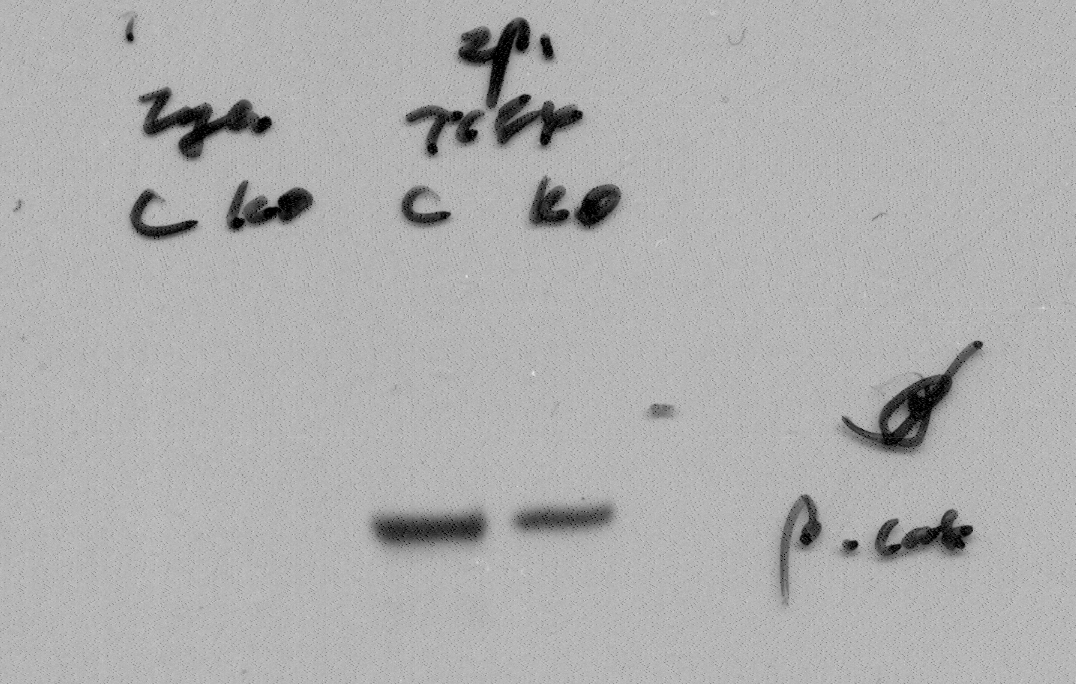

Supplement: Supplementary file 9 — Source data Fig. 4 [file 44318_2024_276_MOESM9_ESM.zip › Figure 4/4H/b-catenin-IP.tif]

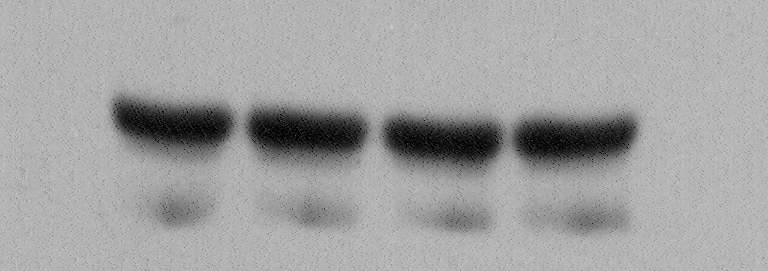

Supplement: Supplementary file 9 — Source data Fig. 4 [file 44318_2024_276_MOESM9_ESM.zip › Figure 4/4H/b-catenin-WCL.tif]

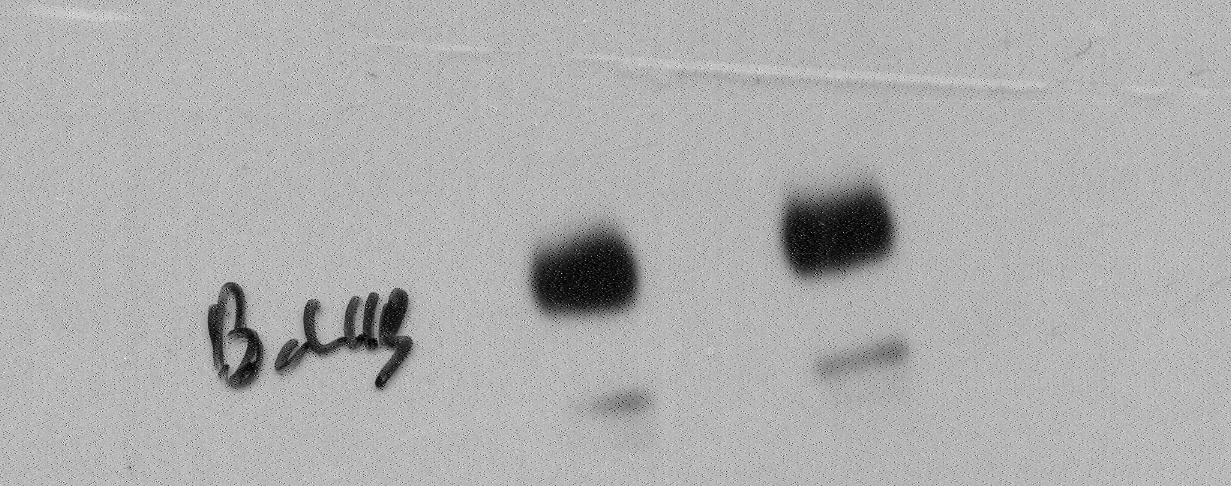

Supplement: Supplementary file 9 — Source data Fig. 4 [file 44318_2024_276_MOESM9_ESM.zip › Figure 4/4H/bcl11b-WCL.tif]

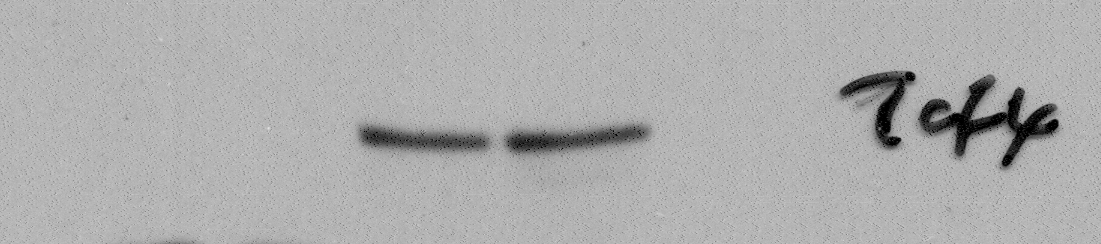

Supplement: Supplementary file 9 — Source data Fig. 4 [file 44318_2024_276_MOESM9_ESM.zip › Figure 4/4H/tcf4-IP.tif]

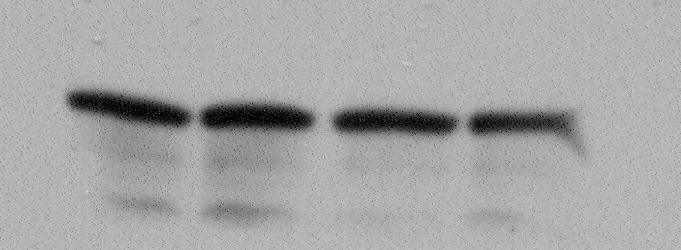

Supplement: Supplementary file 9 — Source data Fig. 4 [file 44318_2024_276_MOESM9_ESM.zip › Figure 4/4H/tcf4-WCL.tif]

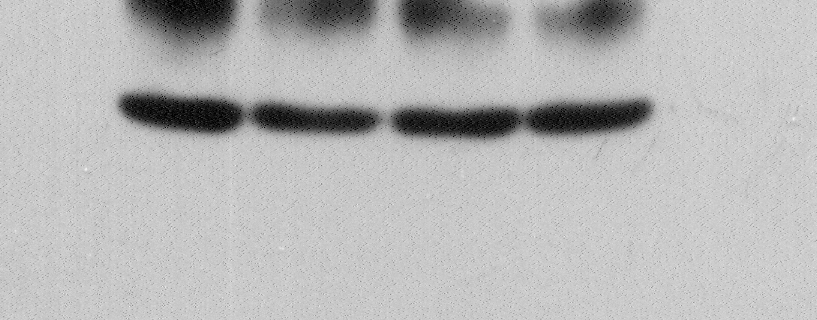

Supplement: Supplementary file 9 — Source data Fig. 4 [file 44318_2024_276_MOESM9_ESM.zip › Figure 4/4H/tubulin.tif]

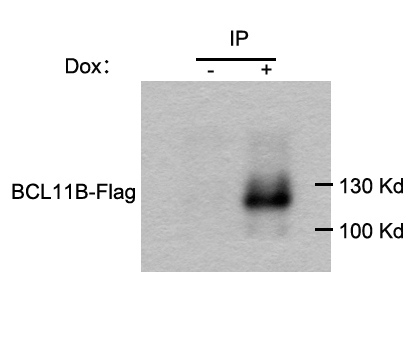

Supplement: Supplementary file 10 — Source data Fig. 5 [file 44318_2024_276_MOESM10_ESM.zip › Figure 5/5B/Bcl11b Flag-IP.tif]

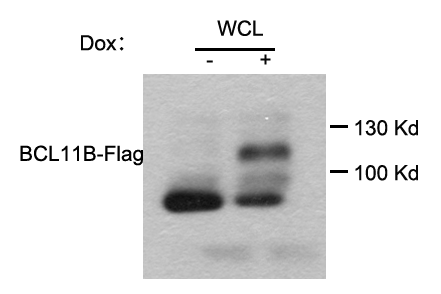

Supplement: Supplementary file 10 — Source data Fig. 5 [file 44318_2024_276_MOESM10_ESM.zip › Figure 5/5B/Bcl11b Flag-WCL.tif]

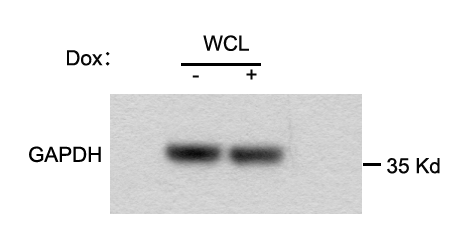

Supplement: Supplementary file 10 — Source data Fig. 5 [file 44318_2024_276_MOESM10_ESM.zip › Figure 5/5B/GAPDH-WCL.tif]

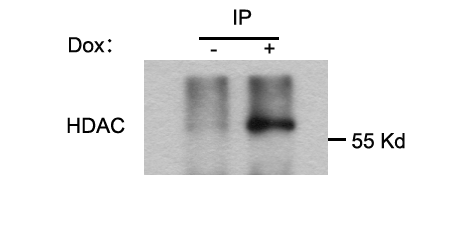

Supplement: Supplementary file 10 — Source data Fig. 5 [file 44318_2024_276_MOESM10_ESM.zip › Figure 5/5B/HDAC1-IP.tif]

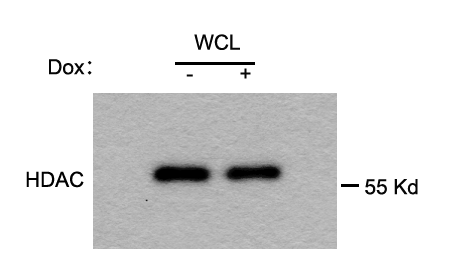

Supplement: Supplementary file 10 — Source data Fig. 5 [file 44318_2024_276_MOESM10_ESM.zip › Figure 5/5B/HDAC1-WCL.tif]

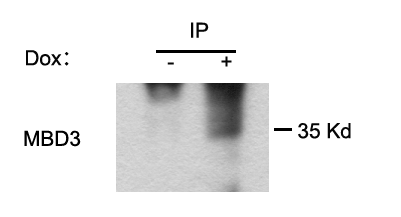

Supplement: Supplementary file 10 — Source data Fig. 5 [file 44318_2024_276_MOESM10_ESM.zip › Figure 5/5B/MBD3-IP.tif]

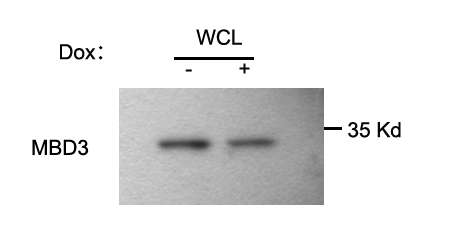

Supplement: Supplementary file 10 — Source data Fig. 5 [file 44318_2024_276_MOESM10_ESM.zip › Figure 5/5B/MBD3-WCL.tif]

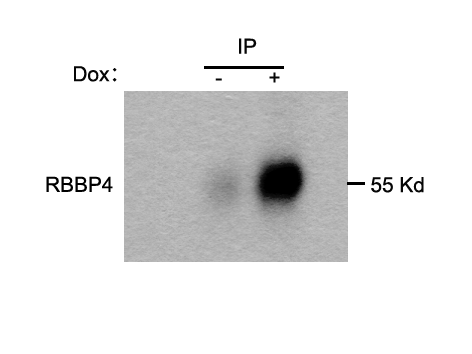

Supplement: Supplementary file 10 — Source data Fig. 5 [file 44318_2024_276_MOESM10_ESM.zip › Figure 5/5B/RBBP4-IP.tif]

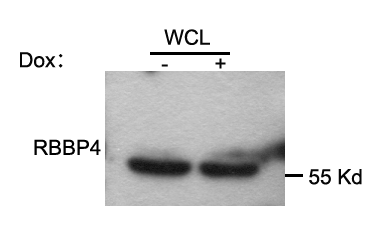

Supplement: Supplementary file 10 — Source data Fig. 5 [file 44318_2024_276_MOESM10_ESM.zip › Figure 5/5B/RBBP4-WCL.tif]
